# Middle Age

**Published:** 1888-09-22

**Authors:** 


### The Doctor's Pulpit.

### MIDDLE AGE.

Lucas Malet introduces us in a recent novel to a heroine of thirty-nine. Not only so, but we fall in love with her before we get half through the book, and remain constant to the very last page. The reader only experiences one regret, that there is no husband worthy of her forthcoming, and she is made an example of the principle which gives the book its title—" A Counsel of Perfection." Is a woman of thirty-nine still young, or is she middle-aged? That depends entirely upon the woman. Lucas Malet's heroine was undoubtedly young at that age, and seemed likely to remain so for many years to come.

Many people have written with more or less wisdom of middle age, but few of them have been quite sure what the period really is. As a rule, middle-aged men and women are not the least interesting to those who are ten, fifteen, or twenty years younger than themselves. The elders do not, perhaps, trouble very much about that. If the man of forty is looked upon as an old fogey by the youth or maiden of twenty he returns the compliment with more than indifference. As a matter of fact, in the great world few men can be very interesting until they have reached the age of forty or more. Until they are so old the world is not able to make up its mind what kind of stuff they are made of. Is the man of forty of common or uncommon mould? Will he fill one of the ten million little round or square holes which are always open to receive the ten million units of commonplace humanity; or will he want room and space specially for himself, and how much room and space will he be able to fill? These are questions which the world often is not able to answer until he approaches middle age; which indeed he is often unable to answer himself.

To some life becomes more and more interesting as middle age approaches: more interesting because larger, more complex, more various in its possibilities, more hopeful in its promises. It is a pity that this should only be true of some. To all the horizon should expand and the way become clearer as life advances. For too many, however, middle age is a period of fixed and hopeless slavery. They have put forth the strength that was in them. That strength has been sufficient to raise them to a certain level. Beyond that they have not the hope and hardly the desire to go. They are now what they must always be; they do what they must always do; life in its aspect of hope and promise is practically closed for them.

The death of hope is the death of effort. " As a man thinks in his heart so is he." Some great authors had not written their first book at forty; some great soldiers had not seen their first battlefield. Nothing is more admirable or more encouraging than to see old men, several of whose names will at once occur to the mind, entering into the conflicts and labours of life with a zeal, energy, and enthusiasm worthy of the period of boyhood. We cannot help thinking they have some secret for preserving the youth of the soul and the vigour of the body. What is the secret? It would be of infinite service, the world thinks, if it could be published abroad to all men. They have a secret no doubt, but it is a secret which, though all men should learn, only some can make use of. To the musician, the works of the great composers are musical; to him who has no ear, they are mere sound. To the man who has in himself an unconquerable spirit, a growing and expanding mind, a courageous versatility, a conviction that all things are possible—to him the examples of old men, strong, hopeful, effective, are new life. But to the drudge, the feeble and hopeless mill-horse, the self-centred and self-seeking, the cowardly and the irresolute, no secret which animates old men, no example which fills the world with admiration, will impart either life or hope. To all men expansion, growing strength, increased effectiveness are possible so long as life and health remain. But though these things are possible to all, it is only the few who attain to them.

It will sound to some like a revelation that the strongest health is only possible to him who is constantly putting forth strong effort. He who makes no effort at all cannot have health. He who merely performs easy routine duties which call for no exercise of conscious energy, soon becomes as tame, as spiritless, and as lifeless as his duties. It is possible and easy for a man to become as feeblebrained and timid as a sheep. When he has reached that stage time often acts like a judicious butcher, and marks him out for killing. Robust effort is necessary to robust health. The more varied the effort the more various will be the developed powers, and the greater will be the health. But to produce great power of mind or body the effort must be real. Mere reading will not make a powerful brain; there must be hand-to-hand conflict with opposing brains as well as the calm pleasures of the study. Similarly there must be strenuous bodily efforts, and they must be put forth daily for a sufficient length of time if bodily power and health are to be the reward. Playing at life, whether physically or intellectually, can only result in the rewards of childhood. It may fill the pockets with intellectual or material marbles, but not with gold. The tin soldier wins no victories, however brilliant a display he may make upon the nursery table.

The majority of men at forty-five and fifty are in the hopeless case of thinking they have seen their best days. There cannot be a more senseless mistake. In some respects the body and mind are different from what they were at twenty-five or thirty. There is no doubt less agility and less readiness to fly here and there upon any and every impulse. But, on the other hand, there should be far more force and staying power, far more experienced skill, and in every way a high degree of practical efficiency. The health should be settled and regular, and the power of resisting disease at its maximum. For all the duties of the period of manhood middle age should be in every sense the fittest and most capable. The man who has sufficient intelligence to see that the meaning of life is work, not play, duty, not pleasure, should hope and expect to retain his practical efficiency until he is seventy or more. This is the way to put off old age to its remotest limit. It is the lazy man who grows old, not the rational worker. It may be true that pensioners live long; that, however, is due to their freedom from care, not to their laziness. They would live still longer, and be a thousand times happier, if their lives were as strenuous as they are sometimes idle and inane.

Middle age, then, is not a period to be regretted by those who have spent youth rationally, and still believe in duty. It is true that old age is within sight, and that experience urges the necessity of doing to-day what the hand finds to do, and of setting about our work at once if we have any great plans and purposes in view. The time for irresolution is past. If a great book is to be written it must now be commenced; if reforms are to be made in the State the work must not be delayed; if faults of habit and character are to be mended they should be taken seriously in hand; if health and strength are to be improved there is no time to be lost. But all these efforts make for health and well-being in every direction. The necessity that is laid upon men to work for every reward which is worth having is of more value than any of the rewards it impels men to obtain. At forty the wise man sees this, and instead of quarrelling with the necessity, puts himself in agreement with it, and not only "reforms his plan," but resolves that he will make it a principle and rule of conduct to keep it reformed in accordance with his improving judgment to the end of life. Looked at thus, middle age. because it is the age of sense and judgment, may, perhaps, be considered the most desirable period of a man's life. He is a sound philosopher who, by the intelligent and well-directed use of all his faculties, makes the period last as long as possible—even to the end of life.

